# Taxonomic revision of the genus Methanobrevibacter, description of Methanomonile shimae gen. nov. sp. nov., and proposal of Methanobrevibacteraceae fam. nov

**DOI:** 10.1099/ijsem.0.007286

**Published:** 2026-09-15

**Authors:** Evgenii Protasov, Mayara Shiratori, Undine S. Mies, Joana Kästle Silva, Thomas Heimerl, Katja Platt, Takashi Itoh, Moriya Ohkuma, Stefan Spring, Aharon Oren, Andreas Brune

**Affiliations:** 1 Research Group Insect Gut Microbiology and Symbiosis, Max Planck Institute for Terrestrial Microbiology, Marburg, Germany; 2 Center for Synthetic Microbiology (SYNMIKRO), Marburg, Germany; 3 Japan Collection of Microorganisms, RIKEN BioResource Research Center, Tsukuba, Japan; 4 Department of Microorganisms, Leibniz Institute DSMZ-German Collection of Microorganisms and Cell Cultures, Braunschweig, Germany; 5 The Institute of Life Sciences, The Hebrew University of Jerusalem, The Edmond J. Safra Campus, Jerusalem, Israel

**Keywords:** *Archaea*, gut microbiota, *Methanobacteriota*, taxonomy

## Abstract

Recent phylogenomic analyses revealed that the genus *Methanobrevibacter*, which consists almost exclusively of representatives from the intestinal tract of animals, is severely underclassified. Based on the large relative evolutionary divergence between individual subclades, members of the genus *Methanobrevibacter* have been reclassified into eight novel genera as new combinations proposed under the rules of the Code of Nomenclature of Prokaryotes Described from Sequence Data. Here, we validly publish the new names for all taxa with type strains also under the rules of the International Code of Nomenclature of Prokaryotes. This includes members of the genera *Methanacia*, *Methanobaculum*, *Methanobinarius*, *Methanocatella* and *Methanoflexus*. Moreover, we propose to place *Methanobrevibacter acididurans*, whose genome was only recently sequenced, in the new genus *Methanobotrus* and describe a new isolate from the gut of a cockroach as *Methanomonile shimae* gen. nov. sp. nov. Based on the large evolutionary distance from the remaining members of *Methanobacteriaceae*, we propose to reclassify all genera within the radiation of *Methanobrevibacter sensu lato* into their own family, *Methanobrevibacteraceae* fam. nov. In addition, we reclassify *Methanothermobacter tenebrarum* as *Methanothermobaculum tenebrarum* gen. nov. comb. nov. into a new family, *Methanothermobaculaceae* (*Methanobacteriales*) and provide emended descriptions for the phylum *Methanobacteriota* and the classes *Methanobacteria* and *Methanococci*.

## Introduction

The genus *Methanobrevibacter* was created to accommodate members of the genus *Methanobacterium* that – based on 16S rRNA oligonucleotide catalogues – formed a separate branch among the few cultures of methanogens available at the time [1]. The genus originally included only *Methanobrevibacter ruminantium*, *Methanobrevibacter arboriphilus* and *Methanobrevibacter smithii*, but during the following years, 13 additional species were described, mostly derived from the intestinal tract of animals. Both 16S rRNA and multi-locus gene sequence analyses of the genus *Methanobrevibacter* indicated the presence of several subclades that apparently coevolved with different host groups (i.e. ruminants, humans and termites) [2, 3]. A comprehensive phylogenomic analysis of the phylum *Methanobacteriota* corroborated this notion and indicated that the genus *Methanobrevibacter* is severely underclassified, requiring the introduction of additional genus-level taxa [4]. Based on all archaeal isolates and metagenome-assembled genomes (MAGs) in the Genome Taxonomy Database (GTDB), Protasov *et al*. [5] presented a taxonomic revision of the *Methanobrevibacter* supergroup, which entailed the proposal of 8 new genera and the reclassification of 12 species. The names were validly published under the Code of Nomenclature of Prokaryotes Described from Sequence Data (SeqCode) [6] based on genomes as type material. However, their validation under the rules of the International Code of Nomenclature of Prokaryotes (ICNP), which requires axenic or defined cultures as type material [7], is still outstanding.

In the present study, we formally describe all taxa with cultured representatives under the rules of the ICNP. In addition, we propose two new genera to accommodate *Methanobrevibacter acididurans* and a new isolate from a cockroach characterized in the present study, whose genomes have recently been sequenced [8]. A comparative analysis of the new genomes with all other species of *Methanobacteriota* demonstrated clear divergence of intestinal *Methanobrevibacter sensu lato* from non-intestinal lineages [8]. Based on the high relative evolutionary divergence (RED) between the taxa in the radiation of *Methanobrevibacter sensu lato* and all other lineages in the family *Methanobacteriaceae*, we propose to reclassify all genera into a separate family, *Methanobrevibacteraceae*. Moreover, we address the polyphyly in the family *Methanothermobacteraceae* by reclassifying *Methanothermobacter tenebrarum* in the genus *Methanothermobaculum* gen. nov. in a new family, *Methanothermobaculaceae* and provide emended descriptions for the phylum *Methanobacteriota* and the classes *Methanobacteria* and *Methanomicrobia*.

## Methods

### Enrichment and isolation

Strain Ec1 was isolated and routinely cultivated in anoxic bicarbonate-buffered mineral medium AM7 supplemented with vitamins and other growth factors [9]. The medium contained (per litre): 1 g NaCl, 0.4 g MgCl_2_·6H_2_O, 0.1 g CaCl_2_·2H_2_O, 0.3 g NH_4_Cl and 0.2 g KH_2_PO_4_; 1 ml trace element solution SL 10 (2 g FeCl_2_·4H_2_O, 70 mg ZnCl_2_, 100 mg MnCl_2_·4H_2_O, 190 mg CoCl2·6H2O, 2 mg CuCl_2_·2H_2_O, 24 mg NiCl_2_·6H_2_O, 36 mg Na_2_MoO_4_·2H_2_O, 6 mg H_3_BO_3_, 5.2 g Na_2_-ethylenediamine-tetraacetate per litre) [10]; 1 ml selenite/tungstate solution (0.5 g NaOH, 3 mg Na_2_SeO_3_·5H_2_O, 4 mg Na_2_WO_4_·2H_2_O per litre); 1 g yeast extract; 1 g Bacto Casamino acids (Gibco); 0.1 g tryptophan (to compensate for the lack of tryptophan in Casamino acids); 1 g casein peptone (Carl ROTH); 1 g sodium acetate; 1 g sodium formate; and 1 ml resazurin solution (0.8 mg l^−1^) as redox indicator. After autoclaving, the medium was cooled under a N_2_/CO_2_ (80 : 20, v/v) atmosphere, and the following supplements (final concentrations) were added from sterile stock solutions, following procedures described by Widdel and Pfennig [11]: NaHCO_3_ (30 mM); 7-vitamin solution (10 mg biotin, 25 mg calcium pantothenate, 50 mg thiamine, 50 mg *p*-aminobenzoic acid, 100 mg nicotinic acid, 250 mg pyridoxamine and 50 mg vitamin B12 per litre) (1 ml l^−1^); menadione (2.5 µM); lipoic acid (1 µM); folic acid and riboflavin (50 µg l^−1^ each); isobutyric, 2-methylbutyric, valeric and isovaleric acids (25 µM each); phenylacetic, phenylpropionic, 4-hydroxyphenylacetic and 3-indolylacetic acids (5 µM each). Cysteine (1 mM) and DTT (1 mM) were added as reducing agents. The pH was adjusted to 7.2 with HCl. The medium was dispensed into 60 ml serum vials (30 ml medium) or 16 ml rubber-stoppered Hungate culture tubes (4.5 ml medium) and gassed with H_2_ + CO_2_ (80 : 20, v/v).

*Ergaula capucina* was taken from a laboratory colony established with specimens previously obtained from a commercial breeder (https://www.schaben-spinnen.de/). An adult female was dissected, and the whole gut was placed into a sterile Hungate tube containing 2-mm glass beads (~2 g). After addition of 10 ml AM7 medium, the tube was closed with a butyl rubber stopper, gassed with N_2_/CO_2_ (80 : 20, v/v) and the gut was homogenized by vortexing for 3 min. The gut homogenate was used to directly inoculate a deep agar dilution series (1% agar) in AM7 medium with vancomycin (100 µg ml^−1^) under a headspace of H_2_ + CO_2_, following standard procedures [12]. After 1 month of incubation at 30 °C, individual colonies were picked with sterile glass pipettes. To ensure clonality of the culture, a second deep-agar dilution series was prepared with culture obtained from the first dilution.

*Methanobrevibacter ruminantium* M1^T^ (DSM 1093), *Methanobrevibacter smithii* PS^T^ (DSM 861), *Methanobrevibacter arboriphilus* DH-1^T^ (DSM 1125) and *Methanobrevibacter cuticularis* RFM-1^T^ (DSM 11139) were obtained from the Deutsche Sammlung von Mikroorganismen und Zellkulturen (DSMZ, Braunschweig, Germany). They were cultivated in the same medium as strain Ec1; in the case of *M. ruminantium* and *M. cuticularis,* the medium was supplemented with clarified rumen fluid (10% v/v).

### Growth, medium requirements and physiology

Growth was measured directly in the culture tubes by following the increase in OD at 578 nm (OD_578_) using a culture tube photometer (Spectronic 20^+^, Milton Roy; path length ca. 1.3 cm). Optimal pH and temperature were determined by comparing methane production under different growth conditions after 7 days of incubation. Dry weight was determined with replicate cultures grown on methanol (50 mM) in 1 l glass vessels containing 500 ml AM7 medium. After OD measurement, the cells were harvested by centrifugation (10,000 *g*; 20 min), washed with ammonium acetate solution (20 mM) and dried at 60 °C until weight constancy.

Substrates tested as alternative electron donors (acetate, formate, propanol and ethanol) were added under a headspace of N_2_/CO_2_ (80 : 20). Methanogenesis from methanol, mono-, di- and trimethylamine, syringate (4-hydroxy-3,5-dimethoxybenzoate), vanillate (4-hydroxy-3-methoxy-benzoate) and 3,4,5-trimethoxybenzoate was tested in phosphate-buffered medium (30 mM) under a headspace of H_2_ or N_2_ [13]. The final concentration of all substrates was 10 mM.

Growth requirements (yeast extract, rumen fluid, coenzyme M, acetate, formate, Casamino acids and vitamins) were assessed after transferring the culture at least three times in medium lacking the respective supplement. A supplement was considered essential if no growth occurred within 2 weeks or as stimulatory if growth and methane production were slower than in full medium.

### Methane measurements

Aliquots of the headspace (0.2 ml) were sampled with a gas-tight syringe (Hamilton), and the methane content was analysed using a GC (SRI) equipped with a packed column (Porapak Q, 80/100 mesh, 274 cm by 3.18 mm inside diameter) and a flame ionization detector.

### Light and electron microscopy

For phase-contrast light microscopy of unfixed cells, an Axiophot photomicroscope (Zeiss, Oberkochen, Germany) was used. For photomicrographs, cultures were mounted on agar-coated glass slides [14]. Autofluorescence of cofactor F_420_ was observed using the same microscope with a UV light source and appropriate filter sets, with *Methanobrevibacter ruminantium* as positive control.

Concentrated cell suspensions (3 µl) were high-pressure frozen, freeze-substituted with an acetone solution containing 0.25% osmium tetroxide, 0.2% uranyl acetate and 5% H_2_O and embedded in Epon 812 substitute resin (for details, see Renicke *et al*. [15]). Ultrathin sections (50 nm) were cut with a microtome equipped with a diamond blade. Sections were post-stained with aqueous solutions of 2% uranyl acetate and 0.5% lead citrate. The sections were examined with a JEM-2100 transmission electron microscope (JEOL, Tokyo, Japan) operated at 120 kV. Images were acquired with a F214 fast-scan CCD camera (TVPIS, Gauting, Germany). Negative-stained images were obtained as described in [16].

### Phylogenetic analyses

The 16S rRNA gene of strain Ec1 was amplified with Archaea-specific primers 109F and 1490R and sequenced as previously described [17]. The sequences were imported into the DictDb 5.0 Archaea reference database [5] using the arb software package (v7.0) [18]. Phylogenetic trees were reconstructed using IQ-TREE 2 (v.2.2.6) with the substitution model GTR+I+G4 [19, 20]. Node support was assessed using ultrafast bootstrap analysis (UFBoot2) and the Shimodaira–Hasegawa approximate-likelihood ratio test (SH-aLRT) [21, 22].

Genomes were classified within the taxonomic framework of the GTDB (release 226). A concatenated and filtered alignment of 53 archaeal single-copy marker genes was generated using the GTDB toolkit (v. 2.4.0) [23], and a maximum-likelihood tree was inferred with IQ-TREE 2 employing the best-fit substitution model selected by ModelFinder [4, 19]. Branch support was assessed with UFBoot2 and the SH-aLRT. For a rank-normalized visualization of the tree, we determined RED values of each node using *PhyloRank* [24]. Taxonomic ranks were harmonized based on the RED values of other taxa with the same rank in the domain *Archaea* [4]. All trees were rendered in arb and edited in Inkscape (v.1.4.2).

## Results and discussion

### Taxonomic revision of the genus *Methanobrevibacter* and proposal of *Methanobrevibacteraceae* fam. nov.

**Fig. 1.**
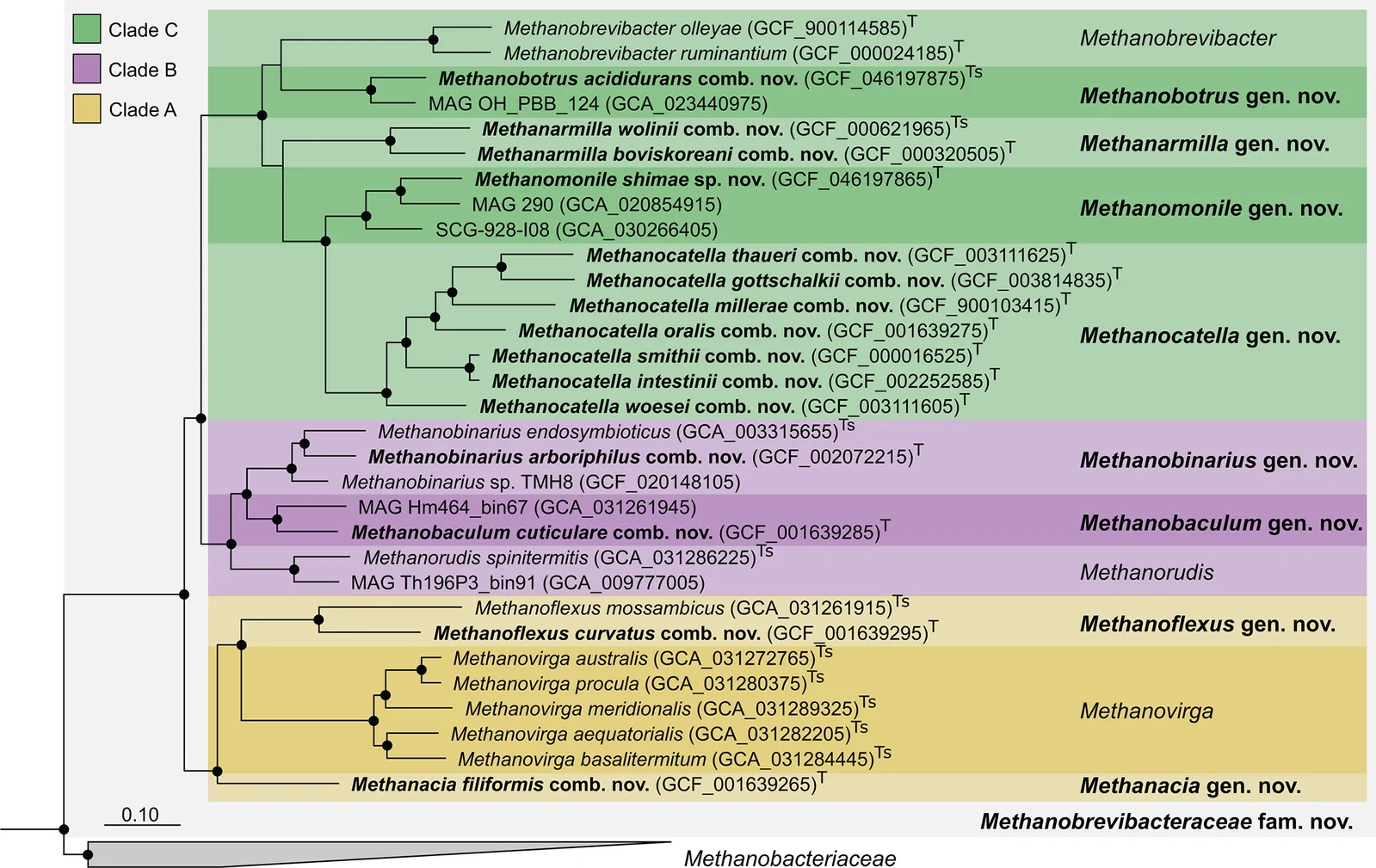
Phylogenomic tree of *Methanobrevibacteraceae* fam. nov. Newly proposed taxa are shown in bold. The index letters indicate whether a species has been described under the ICNP (**T**) or the SeqCode (Ts). The maximum-likelihood tree was inferred from the concatenated alignment of 53 protein-coding genes; bullets on the internal nodes indicate UFBoot support (● ≥99%, 1,000 replicates). The full tree, including all species of *Methanobacteriales,* is shown in the Fig. **S1**.

Phylogenomic analysis of all genomes in the family *Methanobacteriaceae* corroborated the presence of numerous genus-level lineages in the radiation of *Methanobrevibacter sensu lato* (Fig. 1). In addition to the genera proposed already in an earlier study [5], we identified two genus-level lineages that comprise the recently sequenced genomes of *Methanobrevibacter acididurans* and the newly isolated strain Ec1 [8].

The genome of strain Ec1 clusters with a single-cell amplified genome (SCG-928-I08) from the gut of the cockroach *Periplaneta americana* [25] and a metagenome-amplified genome (MAG290) from an anaerobic digester, which are recognized as a genus-level lineage (g__JAHKLT01) in GTDB. Therefore, we propose to classify strain Ec1 as *Methanomonile shimae* gen. nov. sp. nov. The genome of *Methanobrevibacter acididurans* clusters with a second genus-level lineage (g__UBA412) in GTDB, which comprises an MAG from an anaerobic digester (OH_PBB_124). Therefore, we propose to reclassify *Methanobrevibacter acididurans* in the new genus *Methanobotrus*.

With the description of eight genera, nine species and twelve new combinations of existing species (see Protologues section), we conclude our taxonomic revision of the genus *Methanobrevibacter*. At this moment, neither *Methanobotrus acididurans* nor *Methanarmilla wolinii* can be validly published as new combinations under the rules of the ICNP. The type strain of *M. acididurans* ATM^T^ is presently available only from the MACS Collection of Microorganisms in India, a country where proof of availability cannot be issued due to national legislation [26]. Also, the type strain of *Methanobrevibacter wolinii* SH^T^ is presently available from only one culture collection (ATCC). Therefore, we propose the name *Methanobotrus acididurans* under the rules of the SeqCode (the name *Methanarmilla wolinii* has been published already under this code [5]) and propose pro-valid publication of these taxa as *Candidatus* names under the rules of the ICNP [27].

**Fig. 2.**
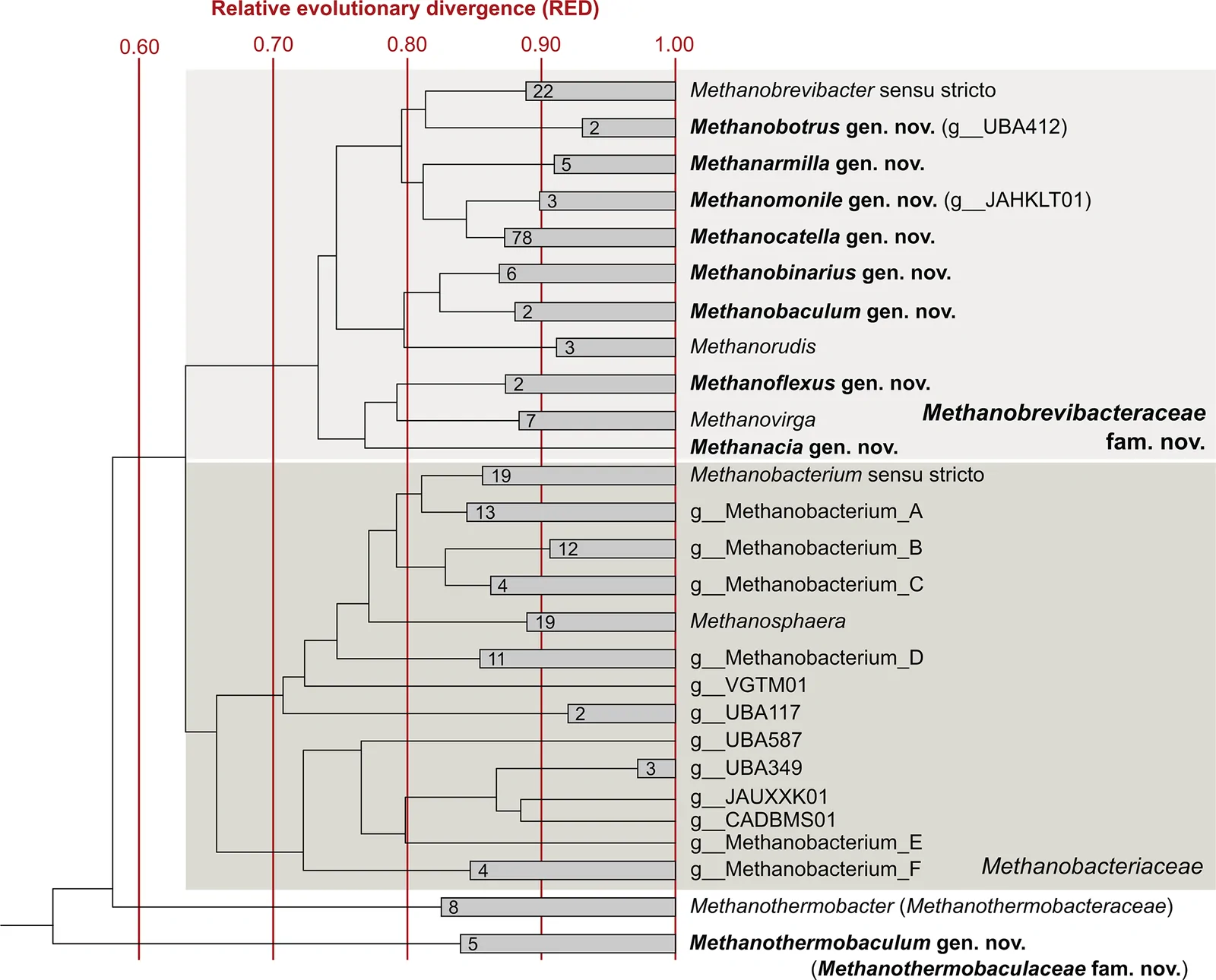
Rank-normalized phylogenies of all genera in the order *Methanobacteriales*. Newly proposed taxa are shown in bold, provisional names in the GTDB taxonomy carry the prefix ‘g__’. The tree is based on the maximum-likelihood tree that was normalized using RED values determined with PhyloRank. The number of genomes in the collapsed clades is indicated. For an expanded version of the original tree, including genome accession numbers, see Fig. S1.

A RED analysis of all type genomes in the phylum *Methanobacteriota*, including all undescribed species recognized in GTDB, confirmed that the RED values of the new genera are in the same range as those of the other genera in the phylum (RED=0.80–0.90; Fig. 2), including the genus *Methanosphaera* and the provisional genus-level lineages in the radiation of the genus *Methanobacterium sensu lato* recognized in GTDB. Moreover, the analysis revealed a deep branch between the lineages in the *Methanobrevibacter sensu lato* clade and the *Methanobacterium*/*Methanosphaera* clade (RED <0.63; Fig. 2), corroborating that the RED of the family *Methanobacteriaceae* is much lower than that of other archaeal families [4]. To harmonize taxonomic ranks among *Methanobacteriota*, we propose to reclassify all genera in the highly supported *Methanobrevibacter sensu lato* clade in the new family *Methanobrevibacteraceae*.

### Reclassification of *Methanothermobacter tenebrarum* and emended descriptions of other taxa

The family *Methanothermobacteraceae* Chuvochina *et al*. 2024 is currently polyphyletic because *Methanothermobacter tenebrarum* Nakamura *et al*. 2013 forms a separate line of descent ([4]; Fig. 1). To resolve this issue, we propose to reclassify this species as *Methanothermobaculum tenebrarum* gen. nov. comb. nov. in the family *Methanothermobaculaceae*. The order *Methanobacteriales* now consists of five family-level lineages: *Methanobacteriaceae, Methanobrevibacteraceae* fam. nov.*, Methanothermaceae*, *Methanothermobacteraceae* and *Methanothermobaculaceae* fam. nov. (Figs 2 and S1, available in the online Supplementary Material).

The phylum name *Methanobacteriota* Garrity and Holt 2023 was proposed as a substitute for ‘*Euryarchaeota*’ Garrity and Holt 2001 by Göker and Oren [28]. Presently, the phylum still contains numerous classes that have been reclassified into other phyla [29], while their nomenclatural types are still assigned to *Methanobacteriota* based on the original description of ‘*Euryarchaeota*’ by Garrity and Holt [30]. This includes *Archaeoglobi* Garrity and Holt 2002, *Halobacteria* Grant *et al*. 2002, *Methanocellia* Chuvochina *et al*. 2024, ‘*Methanomicrobia’* Brenner *et al*. 2005, *Methanonatronarchaeia* Sorokin *et al*. 2018, *Methanosarcinia* Chuvochina *et al*. 2024, and the candidate classes ‘*Candidatus* Hikarchaeia’ Martijn *et al*. 2020, ‘*Candidatus* Methanoliparia’ Borrel *et al*. 2019, ‘*Candidatus* Ordosarchaeia’ Zhao *et al*. 2024’ and ‘*Candidatus* Syntropharchaeia’ Rinke *et al*. 2021, which have been assigned to *Halobacteriota* Chuvochina *et al*. 2024, and *Thermoplasmata* Reysenbach 2002 and ‘*Candidatus* Penumbrarchaeia’ Maeke *et al*. 2025, which have been assigned to *Thermoplasmatota* Chuvochina *et al*. 2024 [31, 32]. *Thermococci* Zillig and Reysenbach 2002 and ‘*Candidatus* Methanofastidiosia’ corrig. Nobu *et al*. 2016 fall into a separate phylum-level lineage (Methanobacteriota_B) [33], which remains to be named and described.

Therefore, we have emended the description of the phylum *Methanobacteriota* by removing all but three classes, specifically *Methanobacteria*, *Methanococci* and *Methanopyri*. In addition, we have emended the description of the class *Methanococci* by excluding the orders *Methanosarcinales* and *Methanomicrobiales*, which belong to the phylum *Halobacteriota*. The descriptions of *Methanobacteria* Boone 2002 and *Methanopyri* Garrity and Hall 2002 remain unchanged. Moreover, we emended the description of the class ‘*Methanomicrobia’* Brenner *et al*. 2005, assigned a type genus and indicated *Halobacteriota* as parent taxon. These taxonomic changes are aimed to achieve a monophyletic status of the phylum *Methanobacteriota*, which share hydrogenotrophic methanogenesis as their major catabolic pathway and separate them from the lineages with a different energy metabolism (methanogenic *Methanosarcinia* and *Methanonatronarchaeia* and non-methanogenic *Archaeoglobi*, *Halobacteria*, *Thermococci* and *Thermoplasmata*).

The protologues of all taxa described or emended in the present study are given below. A list of all species in the phylum *Methanobacteriota* and the class *Methanomicrobia*, including detailed information on taxonomically relevant traits used in the descriptions, is shown in Tables S1 and S2.

### Isolation and characterization of *Methanomonile shimae*

**Fig. 3.**
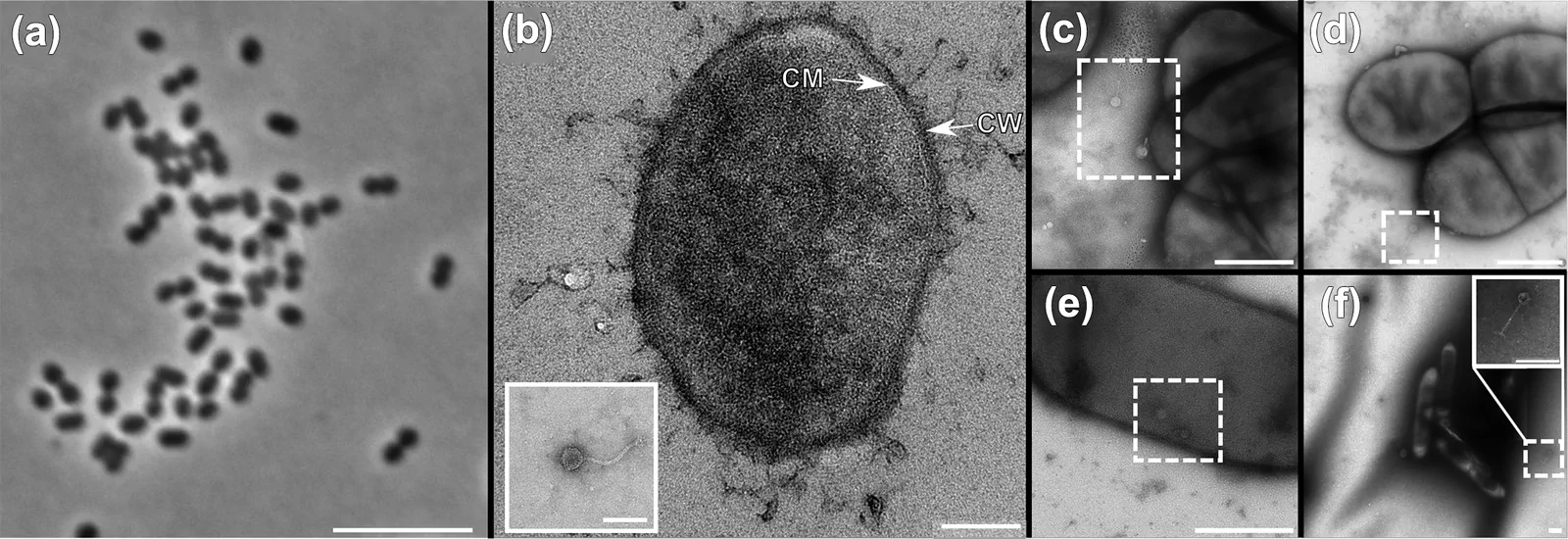
Phase-contrast and electron micrographs of *Methanomonile shimae* (**a, b**), *Methanobrevibacter ruminantium* (**c**), *Methanocatella smithii* (**d**), *Methanobaculum cuticulare* (**e**) and *Methanobinarius arboriphilus* (**f**). The inset in the ultrathin section (**b**) shows a virus from a negative stain of the same culture. Also the boxed regions in c–f (negative stains) indicate virus particles. CM, cytoplasmic membrane; CW, cell wall. Scale bars: 5 µm (**a**), 0.5 µm (b–f), 0.1 µm (insets).

*Methanomonile shimae* strain Ec1 was isolated from a hindgut homogenate of the cockroach *E. capucina*. After serial dilution in deep-agar tubes, the strain formed white lentil-shaped colonies that reach a diameter of up to 2 mm within 2 weeks of incubation at 30 °C (Fig. S2). The cells are coccoid and occur either singly, in pairs or in short chains (Fig. 3). In older cultures, they appear slightly elongated. *Methanomonile shimae* is non-motile, exhibits the characteristic fluorescence of cofactor F_420_ and possesses the cell envelope typical of other *Methanobacteria* (Fig. 3), which is composed of pseudopeptidoglycan, also referred to as archaeal peptidoglycan [34]. Negative stains revealed the presence of viruses in cultures of *Methanomonile shimae*, *Methanobrevibacter ruminantium*, *Methanobinarius arboriphilus* and *Methanobaculum cuticulare* (Fig. 3c–e), which has been observed also in cultures of *Methanocatella smithii* [35]. The viruses are reminiscent of cosmopolitan head-tailed viruses previously found in intestinal *Methanobacteriota* [36] and virus Drs3 infecting *Methanobacterium formicicum* [37].

Strain Ec1 requires H_2_ + CO_2_ as substrates for methanogenesis, using the Wolfe cycle typical of other *Methanobacteriota* [8, 38]. Formate, but not ethanol or propanol, can substitute for H_2_ as electron donor. No methane was formed from acetate, methanol, methylamines or methoxylated aromatic compounds, neither alone nor in the presence of H_2_. Strain Ec1 grew within a temperature range of 20–42 °C, with an optimum at 37 °C. The pH range for growth was 6.5–8.0, with an optimum at pH 7.0–7.5 (Fig. S3). Under optimal conditions (37 °C, pH 7.2, growth yield on H_2_ + CO_2_ was 1.3 g dry mass mol^−1^ of methane). The corresponding doubling time was 14.7 h (Table 1). Acetate, amino acids, vitamins and yeast extract were required for growth. Growth was not inhibited by kanamycin, ampicillin or vancomycin (final concentrations 100 µg ml^−1^). However, no growth was observed in the presence of chloramphenicol (10 µg ml^−1^). The description of *Methanomonile shimae* Ec1 follows minimal standards for the description of methanogenic archaea [39].

**Table 1.** Phenotypic properties of the type species of all genera in *Methanobrevibacteraceae* fam. nov.

Species and type strain	*Methanobrevibacter ruminantium* M1^T^	*Methanomonile shimae* Ec1^T^	*Methanarmilla wolinii* JH-1^T^	*Methanocatella smithii* PS^T^	*Methanobotrus acididurans* ATM^T^	*Methanobinarius arboriphilus* DH-1^T^	*Methanobaculum cuticulare* RFM-1^T^	*Methanoﬂexus curvatus* RFM-2^T^	*Methanacia filiformis* RFM-3^T^
Isolation source	Cow rumen	Cockroach gut	Sheep rumen	Sewage sludge	Anaerobic digester	Wetwood	Termite gut	Termite gut	Termite gut
Cell morphology	Coccoid rods or short oval rods, occur in pairs or in chains	Cocci, occur singly, in pairs or in chains	Coccoid rods, occur in pairs or in short chains	Coccoid rods or short oval rods, occur singly, in pairs or in chains	Cocci, occur singly, in pairs, or in chains	Short rods, occur singly or in pairs	Short rods, occur singly, in pairs or in short chains	Curved rods, occur singly or in pairs	Filament-forming rods
Cell size (diameter/length) (µm)	0.7/0.8–1.7	0.5–2.0	0.6/1.0–1.4	0.5–1.0/1.0–1.5	0.3–0.5	0.5/1.2–1.4	0.4–1.2	0.34/1.6	0.23–0.28/up to several hundred
Energy substrates*									
H_2_ + CO_2_	+	+	+	+	+	+	+	+	+
Formate	+	+	– †	+	– †	– †	w	– †	– †
H_2_+methanol	–	ND†	ND†	ND†	–	ND†	–	–	–
Growth factors‡									
Acetate	+	+	+	+	+	–	–	+	–
Yeast extract	–	+	+	–	–	–	s	–	+
Rumen fluid	+	–	–	–	+	–	s	+	–
Coenzyme M	+	–	–	–	–	–	–	–	–
Amino acids	+	+	–	–	+	–	–	–	–
Vitamins	+	+	–	+	–	+	ND	–	–
Temp. optimum (range) (°C)	ND	37 (25–42)	37	37–39	35 (25–37)	30–37 (10–45)	37 (10–42)	30 (10–37)	30 (10–33.5)
pH optimum (range)	ND	7.0–7.5 (6.5–8)	7	6.9–7.4	6.0 (5.0–7.5)	7.5–8	7.7 (6.5–8.5)	7.1–7.2 (6.5–8.5)	7.0–7.2 (6.0–7.5)
Genome size (Mbp)	2.96	1.93	2.04	1.85	1.70	2.45	2.67	2.47	2.71
G+C content (mol%)	32.6	26.4	24.2	31.0	27.4	25.4	26.8	25.7	27.0

* +, growth; –, no growth; w, weak growth.

† Pathway encoded in genome.

‡ +, essential; –, not required.

nd, not determineds, stimulatory.

**Fig. 4.**
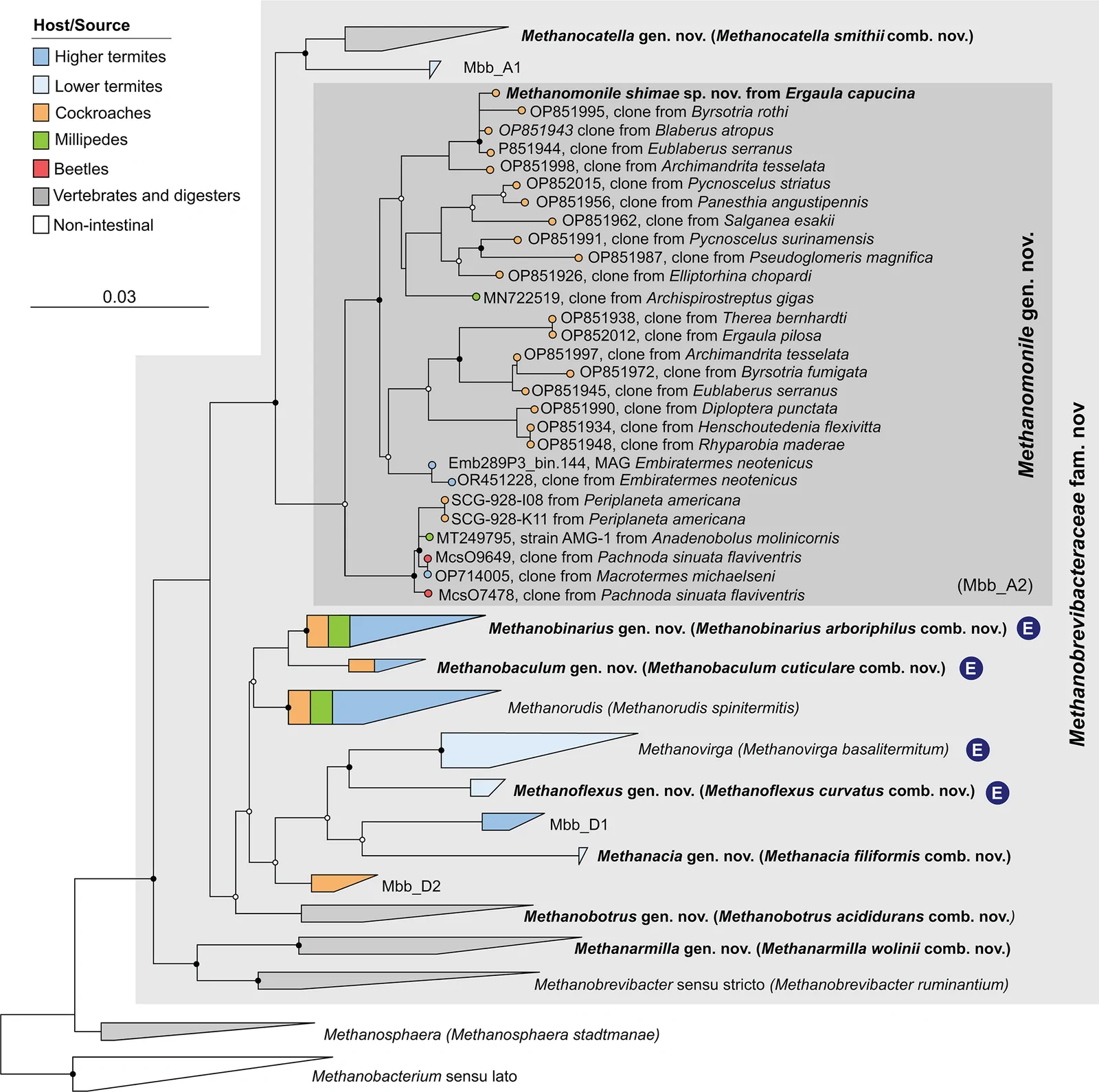
16S rRNA gene tree of *Methanobrevibacteraceae* fam. nov., showing the host range of individual genera. Newly proposed taxa are shown in bold. Type species are given in parentheses. The genus *Methanomonile* has been expanded. Clades with putative endosymbionts (**e**) are marked. The maximum-likelihood tree is based on a curated alignment of near-full-length 16S rRNA genes (>1,400 sites) and was generated using IQ-TREE under the GTR+I+G4 model of evolution. Bullets on the internal nodes indicate UFBoot support (● ≥95%, 1,000 replicates). The scale bar indicates the number of substitutions per site. The tree was rooted with other representatives of *Methanobacteriales*.

Phylogenetic analysis placed the 16S rRNA gene sequence of strain Ec1, together with its homologs from the single-cell amplified genomes from *P. americana* SCG-928-I08 and SCG-928-K11 [25], within a clade harbouring numerous uncultured archaea from other cockroaches and millipedes (Fig. 4). This links the genus *Methanomonile*, which has been defined based on phylogenomic analysis (Fig. 1), with the 16S rRNA genes of the Mbb_A2 clade, which so far lacked representatives with genomic data [5]. In both trees, the genus *Methanomonile* occupies a sister position to the genus *Methanocatella*, which consists exclusively of representatives from mammals and birds. Three clades of *Methanobrevibacteraceae* with representatives from lower termites (Mbb_A1), higher termites (Mbb_D1) and cockroaches (Mbb_D2) remain without sequenced genomes.

## Protologues

### *Methanobacteriota* Garrity and Holt 2023, emend. Protasov and Brune

**Etymology:** *Methanobacteriota* (Me.tha.no.bac.te.ri.o'ta. N.L. neut. n. *Methanobacterium*, type genus of the phylum; L. neut. pl. suff. *-ota*, suffix to denote a phylum; N.L. neut. pl. n. *Methanobacteriota*, the phylum of the genus *Methanobacterium*).

**Description:** Members of the phylum form a monophyletic group defined by both phylogenomic analysis and its relative evolutionary divergence from other members of the kingdom *Methanobacteriati*. Cell walls are composed of pseudopeptidoglycan (archaeal peptidoglycan) and/or a proteinaceous S-layer. Strictly anaerobic. Conserve energy by methanogenesis via the Wolfe cycle, typically from H_2_ + CO_2_. Members of the phylum are present in most anoxic habitats. The phylum consists of the classes *Methanobacteria*, *Methanococci* and *Methanopyri*.

**Type genus:** *Methanobacterium* Kluyver and van Niel (Approved Lists 1980)

**Parent taxon:** *Methanobacteriota* (Garrity and Holt 2023) Oren and Göker 2024

### *Methanococci* Boone 2002, emend. Protasov and Brune

**Etymology:** *Methanococci* (Me.tha.no.coc'ci. N.L. masc. n. *Methanococcus*, type genus of the class; N.L. masc. pl. n. *Methanococci*, the class of the genus *Methanococcus*).

**Description:** Members of the class form a monophyletic group defined by both phylogenomic analysis and its relative evolutionary divergence from other members of the phylum *Methanobacteriota*. Cells are regular or irregular cocci, occur singly or in pairs. Cell walls are composed of a proteinaceous S-layer; pseudopeptidoglycan (archaeal peptidoglycan) is absent. Cells stain Gram-negative. Most species are motile. Archaella are typically organized in tufts. Strictly anaerobic. Use H_2_ + CO_2_ as substrate for methanogenesis; some species also use formate as electron donor. (Hyper)thermophilic or mesophilic. All species were isolated from marine environments, including deep and shallow sediments and deep-water hydrothermal vents. The class has only one order, *Methanococcales*.

**Type genus:** *Methanococcus* Kluyver and van Niel (Approved Lists 1980)

**Parent taxon:** *Methanobacteriota* Garrity and Holt 2023, emend. Protasov and Brune

### *Methanomicrobia* Brenner *et al*., emend. Protasov and Brune

**Etymology:** Me.tha.no.mi.cro'bi.a. N.L. neut. n. *Methanomicrobium*, type genus of the class; L. neut. pl. n. suff. *-ia*, ending to denote a class; N.L. neut. pl. n. *Methanomicrobia*, the class of the genus *Methanomicrobium*.

**Synonyms:** ‘*Methanomicrobia*’ Garrity *et al*. 2003 (not validly published); ‘*Methanomicrobia*’ Brenner *et al*. 2005 (not validly published).

**Description:** Members of the class form a monophyletic group defined by both phylogenomic analysis and its relative evolutionary divergence from other members of the phylum *Halobacteriota*. Cells are regular or irregular cocci. Cell walls are composed of a proteinaceous S-layer. Cells stain predominantly Gram-negative. Strictly anaerobic. Use H_2_ + CO_2_ as substrates for methanogenesis; some species also use formate, 2-propanol or 2-butanol as electron donor. Predominantly mesophilic. Widely distributed in anoxic habitats, including soils, sediments, the intestinal tract of various animals and anaerobic sewage digesters. The class has two orders, *Methanomicrobiales* and ‘*Candidatus* Methanoflorentales’.

**Type genus:** *Methanomicrobium* Balch and Wolfe 1981

**Parent taxon:** Halobacteriota Chuvochina *et al*. 2024

### *Methanobrevibacteraceae* Protasov and Brune fam. nov.

**Etymology:** *Methanobrevibacteraceae* (Me.tha.no.bre.vi.bac.te.ra'ce.ae. N.L. masc. n. *Methanobrevibacter*, type genus of the family; *-aceae* ending to denote a family; N.L. fem. pl. n. *Methanobrevibacteraceae*, the family of the genus *Methanobrevibacter*).

**Description:** Members of the family form a monophyletic group defined by both phylogenomic analysis and its relative evolutionary divergence from other members of the order *Methanobacteriales*. Cells are rods, filaments or regular or irregular cocci. Cell walls are composed of pseudopeptidoglycan (archaeal peptidoglycan). Cells stain predominantly Gram-positive. Non-motile. Strictly anaerobic. Use H_2_ + CO_2_ as substrates for methanogenesis; some species also use formate as electron donor. Mesophilic, with optimum growth temperature of 35–45 °C. Most species occur in the intestinal tract of animals. Most species are neutrophiles; pH optimum 6.0–8.0. Genome size ranges from 1.54 to 3.49 Mbp; G+C content from 24.2 to 41.2 mol%. The family comprises the genera *Methanobrevibacter, Methanacia, Methanarmilla, Methanobaculum, Methanobinarius, Methanobotrus, Methanocatella, Methanoflexus, Methanomonile*, *Methanorudis* and *Methanovirga*.

**Type genus:** *Methanobrevibacter* Balch and Wolfe 1981

**Parent taxon:** *Methanobacteriales* Balch and Wolfe 1981

### *Methanobrevibacter* Balch and Wolfe 1981, emend. Protasov and Brune

**Etymology:** Me.tha.no.bre.vi.bac'ter. N.L. neut. n. *methanum*, methane; N.L. pref. *methano-*, pertaining to methane; L. masc. adj. *brevis*, short; N.L. masc. n. *bacter*, rod, staff; N.L. masc. n. *Methanobrevibacter*, short methane-producing rod.

**Description:** Members of the genus form a monophyletic group defined by both phylogenomic analysis and its relative evolutionary divergence from other members of the family *Methanobrevibacteraceae*. Cells are rods, 0.3–1 µm in diameter and up to 1.7 µm in length; they occur singly, in pairs or in chains. Cells stain Gram-positive. Non-motile. Strictly anaerobic. Use H_2_ + CO_2_ as substrates for methanogenesis; some species also use formate as electron donor. Require complex media supplemented with yeast extract and acetate. Optimum growth temperature is 35–42 °C. Genome size ranges from 2.12 to 2.96 Mbp; G+C content from 26.8 to 32.6 mol%.

**Type species:** *Methanobrevibacter ruminantium* (Smith and Hungate 1958) Balch and Wolfe 1981

**Parent taxon:** *Methanobrevibacteraceae* Protasov and Brune

### *Methanacia* Protasov and Brune gen. nov.

**Etymology:** Me.than.a'ci.a. N.L. neut. n. *methanum*, methane; N.L. pref. *methano-*, pertaining to methane; L. fem. n. *acia*, thread, yarn; N.L. fem. n. *Methanacia*, a methane-producing thread.

**Synonym:** *‘Methanacia’* Protasov and Brune 2023 (not validly published).

**Description:** Members of the genus form a monophyletic group defined by both phylogenomic analysis and its relative evolutionary divergence from other members of the family *Methanobrevibacteraceae*. Cells are filamentous rods with slightly tapered ends. Cells stain Gram-positive. Non-motile. Strictly anaerobic. Use H_2_ + CO_2_ as substrates for methanogenesis. Optimum growth temperature is 30 °C. Require media supplemented with yeast extract. Genome size ranges from 2.35 to 2.71 Mbp; G+C content from 25.3 to 27.0 mol%.

**Type species**: *Methanacia filiformis*

**Parent taxon:** *Methanobrevibacteraceae* Protasov and Brune

### *Methanacia filiformis* (Leadbetter *et al*. 1998) Protasov and Brune comb. nov.

**Etymology:** fi.li.for'mis. L. neut. n. *filum*, a thread; L. masc./fem. adj. suff. *-formis*, -like, in the shape of; from L. fem. n. *forma*, figure, shape, appearance; N.L. fem. adj. *filiformis*, thread-shaped.

**Basonym:** *Methanobrevibacter filiformis* Leadbetter *et al*. 1998

**Synonym:** *‘Methanacia filiformis’* Protasov and Brune 2023 (not validly published).

**Description:** The species description remains the same as in Miller [40]. Includes all genomes with ≥95% average nucleotide identity to the type genome. The type strain has a genome size of 2.6 Mbp and a G+C content of 27.0 mol%.

**Type strain:** RFM-3^T^ (DSM 11501, JCM 39454) isolated from the termite *Reticulitermes flavipes*.

**GenBank accession number:** GCF_001639265 (genome); U82322 (16S rRNA).

### *Methanarmilla* Protasov and Brune gen. nov.

**Etymology:** Me.than.ar.mil'la. N.L. neut. n. *methanum*, methane; N.L. pref. *methano-*, pertaining to methane; L. fem. n. *armilla*, bracelet; N.L. fem. n. *Methanarmilla*, methane-producing bracelet, referring to the short chains of cells formed by the type species.

**Synonym:** *‘Methanarmilla*’ Protasov and Brune 2023 (not validly published).

**Description:** Members of the genus form a monophyletic group defined by both phylogenomic analysis and its relative evolutionary divergence from other members of the family *Methanobrevibacteraceae*. Cells are short oval rods or coccobacilli, 0.6 µm in width and 1–1.8 µm in length and occur singly, in pairs or in chains. Cells stain Gram-positive. Non-motile. Strictly anaerobic. Use H_2_ + CO_2_ as substrate for methanogenesis. Optimum growth temperature is 37–40 °C. Require complex media supplemented with acetate, yeast extract, trypticase, volatile fatty acids and coenzyme M. Genome size is ~2.0 Mbp; G+C content ranges from 24.2 to 29.0 mol%.

**Type species:** *Methanarmilla wolinii*

**Parent taxon:** *Methanobrevibacteraceae* Protasov and Brune

### *Methanarmilla wolinii* (Miller and Lin 2002) Protasov and Brune comb. nov.

**Etymology:** wo.lin'i.i. N.L. gen. masc. n. *wolinii*, of Wolin, named in honour of Meyer J. Wolin for his singular contributions to the physiological understanding of the role of methanogens and interspecies hydrogen transfer in anaerobic habitats.

**Basonym:** *Methanobrevibacter wolinii* Miller and Lin 2002

**Synonym:** *‘Methanarmilla wolinii’* Protasov and Brune 2023 (not validly published).

**Description:** The species description remains the same as in Miller and Lin [41]. Includes all genomes with ≥95% average nucleotide identity to the genome of the type strain. The type strain has a genome size of 2.0 Mbp and a G+C content of 24.2 mol%.

**Type strain:** SH^T^ (ATCC BAA-1170) isolated from sheep faeces. The strain is no longer available at DSMZ (DSM 11976).

**GenBank accession number:** GCF_000621965 (genome); U55240 (16S rRNA).

### *Methanarmilla boviskoreani* (Lee *et al*. 2013) Protasov and Brune comb. nov.

**Etymology:** bo.vis.ko.re.a'ni. L. masc./fem. n. *bos*, cattle; N.L. masc. adj. *koreanus*, Korean; N.L. gen. n. *boviskoreani*, of Korean cattle.

**Basonym:** *Methanobrevibacter boviskoreani* Lee *et al*. 2013.

**Synonym:** *‘Methanarmilla boviskoreani’* Protasov and Brune 2023 (not validly published).

**Description:** The species description remains the same as in Lee *et al*. [42]. Includes all genomes with ≥95% average nucleotide identity to the genome of the type strain. The type strain has a genome size of 2.0 Mbp and a G+C content of 28.9 mol%.

**Type strain:** JH-1^T^ (DSM 25824, JCM 18376, KCTC 4102) isolated from the rumen of Korean native cattle (*Bos taurus coreanae*).

**GenBank accession number:** GCF_000320505 (genome); KC608769 (16S rRNA).

### *Methanobaculum* Protasov and Brune gen. nov.

**Etymology:** Me.tha.no.ba'cu.lum. N.L. neut. n. *methanum*, methane; N.L. pref. *methano-*, pertaining to methane; L. neut. n. *baculum*, small rod; N.L. neut. n. *Methanobaculum*, a small methane-producing rod.

**Synonym:** *‘Methanobaculum’* Protasov and Brune 2023 (not validly published).

**Description:** Members of the genus form a monophyletic group defined by both phylogenomic analysis and its relative evolutionary divergence from other members of the family *Methanobrevibacteraceae*. Cells are short oval rods with slightly tapered ends that occur singly, in pairs, or in short chains. Cells stain Gram-positive. Non-motile. Strictly anaerobic. Use H_2_ + CO_2_ as substrates for methanogenesis; grow poorly on formate. Optimum growth temperature is 30–37 °C. Grow poorly on mineral medium with vitamins; addition of yeast extract, Casamino acids and rumen fluid strongly stimulates growth. Genome size ranges from 1.40 to 2.67 Mbp; G+C content from 25.6 to 26.8 mol%.

**Type species:** *Methanobaculum cuticulare* corrig.

**Parent taxon:** *Methanobrevibacteraceae* Protasov and Brune

### *Methanobaculum cuticulare* corrig. (Leadbetter and Breznak 1997) Protasov and Brune comb. nov.

**Etymology:** cu.ti.cu.la're. L. fem. n. *cuticula*, a thin skin, cuticle; N.L. neut. adj. *cuticulare*, referring to the cuticular surface of its habitat, the hindgut epithelium of termites.

**Basonym:** *Methanobrevibacter cuticularis* Leadbetter and Breznak 1997.

**Synonym:** *‘Methanobaculum cuticulare’* Protasov and Brune 2023 (not validly published).

**Description:** The species description remains the same as in Miller [40]. Includes all genomes with ≥95% average nucleotide identity (ANI) to the type genome. The type strain has a genome size of 2.6 Mbp and a G+C content of 26.8 mol%.

**Type strain:** RFM-1^T^ (DSM 11139, JCM 39453) isolated from the termite *Reticulitermes flavipes*.

**GenBank accession number:** GCA_001639285 (genome); U41095 (16S rRNA).

### *Methanobinarius* Protasov and Brune gen. nov.

**Etymology:** Me.tha.no.bi.na'ri.us. N.L. neut. n. *methanum*, methane; N.L. pref. *methano-*, pertaining to methane; L. masc. adj. *binarius*, consisting of two things; N.L. masc. n. *Methanobinarius*, methane-producing (organism) consisting of two things, referring to the pairs of cells formed by the type species.

**Synonym:** *‘Methanobinarius’* Protasov and Brune 2023 (not validly published).

**Description:** Members of the genus form a monophyletic group defined by both phylogenomic analysis and its relative evolutionary divergence from other members of the family *Methanobrevibacteraceae*. Cells are short oval rods, 0.5–0.75 µm in diameter and 1.8–3.5 µm in length; they occur singly, in pairs or in chains. Cells stain Gram-positive. Non-motile. Strictly anaerobic. Use H_2_ + CO_2_ as substrates for methanogenesis. Optimum growth temperature is 37–40 °C. Grow poorly on mineral medium with vitamins; addition of yeast extract, casamino acids and rumen fluid strongly stimulates growth. Genome size ranges from 1.9 to 2.0 Mbp; G+C content from 24.2 to 25.3 mol%.

**Type species:** *Methanobinarius arboriphilus*

**Parent taxon:** *Methanobrevibacteraceae* Protasov and Brune

### *Methanobinarius arboriphilus* (Zeikus and Henning 1975) Protasov and Brune comb. nov.

**Etymology:** ar.bo.ri'phi.lus. L. fem. n. arbour, tree; N.L. masc. adj. suff. *-philus*, friend, loving; from Gr. masc. adj. *philos*; N.L. masc. adj. *arboriphilus*, tree-loving.

**Basonym:** *Methanobrevibacter arboriphilus* corrig. (Zeikus and Henning 1975) Balch and Wolfe 1981.

**Synonyms:** *Methanobrevibacter arboriphilicus* (Zeikus and Henning 1975) Balch and Wolfe 1981; *Methanobacterium arbophilicum* Zeikus and Henning 1975 (Approved Lists 1980); *‘Methanobinarius arboriphilus’* Protasov and Brune 2023 (not validly published).

**Description:** The species description remains the same as in Miller [40]. Includes all genomes with ≥95% average nucleotide identity to the genome of the type strain. The type strain has a genome size of 2.0 Mbp and a G+C content of 24.2 mol%.

**Type strain:** DH1^T^ (ATCC 33747, DSM 1125, JCM 13429) isolated from wetwood cores.

**GenBank accession number:** GCF_002072215 (genome); AY196665 (16S rRNA).

### *Methanobotrus* Protasov and Brune gen. nov.

**Etymology:** Me.tha.no.bo'trus. N.L. neut. n. *methanum*, methane; N.L. pref. *methano-*, pertaining to methane; L. fem. n. *botrus*, a bunch of grapes; N.L. fem. n. *Methanobotrus*, a methane-producing cluster of cells resembling a bunch of grapes.

**Description:** Members of the genus form a monophyletic group defined by both phylogenomic analysis and its relative evolutionary divergence from other members of the family *Methanobrevibacteraceae*. Cells are cocci; they occur singly, in pairs or in chains. Non-motile. Strictly anaerobic. Use H_2_ + CO_2_ as substrates for methanogenesis. Require complex medium supplemented with rumen fluid, acetate and amino acids. Genome size ranges from 1.68 to 1.84 Mbp; G+C content from 27.2 to 27.9 mol%.

**Type species:** *Methanobotrus acididurans*

**Parent taxon:** *Methanobrevibacteraceae* Protasov and Brune

### *Methanobotrus acididurans* (Savant *et al*. 2002) Protasov and Brune comb. nov.

**Etymology:** aci.di.du'rans. L. neut. adj. *acidum*, an acid; from L. masc. adj. *acidus*, sour; L. pres. part. *durans*, resisting; N.L. masc. part. adj. *acididurans*, acid-resisting.

**Basonym:** *Methanobrevibacter acididurans* Savant *et al*. 2002.

**Description:** Cells are cocci, 0.3–0.5 µm in diameter; they occur singly, in pairs or in chains. Cells stain Gram-positive and are resistant to lysis with SDS and in hypotonic solutions. Non-motile. Strictly anaerobic. Require yeast extract or rumen fluid for growth. Optimum growth temperature is 35 °C. The pH optimum is 6.0. Use H_2_ + CO_2_ as substrates for methanogenesis. Includes all genomes with ≥95% average nucleotide identity to the genome of the type strain. The type strain has a genome size of 1.70 Mbp and a G+C content of 27.4 mol%.

**Type strain:** ATM^T^ (MCM-A-613) isolated from an anaerobic digester. The strain is no longer available at DSMZ (DSM 15163).

**GenBank accession number:** GCF_046197875 (genome); AF242652 (16S rRNA).

### *Methanocatella* Protasov and Brune gen. nov.

**Etymology:** Me.tha.no.ca.tel'la. N.L. neut. n. *methanum*, methane; N.L. pref. *methano-*, pertaining to methane; L. fem. n. *catella*, an ornamental chain; N.L. fem. n. *Methanocatella*, a methane-producing chain, referring to the short chains of cells characteristic of this genus.

**Synonym:** *‘Methanocatella*’ Protasov and Brune 2023 (not validly published).

**Description:** Members of the genus form a monophyletic group defined by both phylogenomic analysis and its relative evolutionary divergence from other members of the family *Methanobrevibacteraceae*. Cells are short oval rods or coccobacilli, 0.4–1 µm in width and 0.6–1.5 µm in length. Cells occur singly, in pairs or in chains. Cells stain Gram-positive. Non-motile. Strictly anaerobic. Use H_2_ + CO_2_ as substrates for methanogenesis; some species grow poorly on formate. Optimum growth temperature is 35–42 °C. Require complex media supplemented with yeast extract, trypticase, rumen fluid or faecal extract. Genome size ranges from 1.54 to 2.73 Mbp; G+C content from 27.7 to 36.9 mol%.

**Type species:** *Methanocatella smithii*

**Parent taxon:** *Methanobrevibacteraceae* Protasov and Brune

### *Methanocatella smithii* (Balch and Wolfe 1981) Protasov and Brune comb. nov.

**Etymology:** smith'i.i. N.L. gen. masc. n. *smithii*, of Smith, named after P.H. Smith, who isolated the type strain.

**Basonym:** *Methanobrevibacter smithii* Balch and Wolfe 1981.

**Synonym:** *‘Methanocatella smithii’* Protasov and Brune 2023 (not validly published).

**Description:** The description remains the same as in Miller [40]. Includes all genomes with≥95% average nucleotide identity to the genome of the type strain. The type strain has a genome size of 1.85 Mbp and a G+C content of 31.0 mol%.

**Type strain:** PS^T^ (DSM 861, JCM 30028) isolated from a sewage digester.

**GenBank accession number:** GCF_000016525 (genome); U55233 (16S rRNA).

### *Methanocatella intestini* (Weinberger *et al*. 2025) Protasov and Brune comb. nov.

**Etymology:** in.tes.ti'ni. L. gen. n. *intestini*, of the gut.

**Basonym:** *Methanobrevibacter intestini* Weinberger *et al*. 2025.

**Description:** The description remains the same as in Weinberger *et al*. 2025 [43]. Includes all genomes with ≥95% average nucleotide identity to the genome of the type strain. The type strain has a genome size of 1.92 Mbp and a G+C content of 30.3 mol%.

**Type strain:** WWM1085^T^ (DSM 116060, CECT 30992) isolated from human faeces.

**GenBank accession number:** GCF_002252585.1 (genome); PQ686616 (16S rRNA).

### *Methanocatella gottschalkii* (Miller and Lin 2002) Protasov and Brune comb. nov.

**Etymology:** gott.schalk'i.i. N.L. gen. masc. n. *gottschalkii*, of Gottschalk, named in honour of Gerhard Gottschalk for his notable contributions to the understanding of the biochemistry of methanogenesis.

**Basonym:** *Methanobrevibacter gottschalkii* Miller and Lin 2002.

**Synonym:** *‘Methanocatella gottschalkii’* Protasov and Brune 2023 (not validly published).

**Description:** The description remains the same as in Miller and Lin [41]. Includes all genomes with ≥95% average nucleotide identity to the genome of the type strain. The type strain has a genome size of 1.87 Mbp and a G+C content of 30.0 mol%.

**Type strain:** HO (ATCC BAA-1169, DSM 11977) isolated from horse faeces.

**GenBank accession number:** GCF_003814835 (genome); U55238 (16S rRNA).

### *Methanocatella millerae* (Rea *et al*. 2007) Protasov and Brune comb. nov.

**Etymology:** mil'le.rae. N.L. gen. fem. n. *millerae*, of Miller, named after Terry L. Miller for her contributions to the taxonomy of methanogens, in particular the genus *Methanobrevibacter*.

**Basonym:** *Methanobrevibacter millerae* Rea *et al*. 2007.

**Synonym:** *‘Methanocatella millerae’* Protasov and Brune 2023 (not validly published).

**Description:** The description remains the same as in Rea *et al*. [44]. Includes all genomes with ≥95% average nucleotide identity (ANI) to the genome of the type strain. The type strain has a genome size of 2.72 Mbp and a G+C content of 36.5 mol%.

**Type strain:** ZA-10^T^ (DSM 16643, JCM 39457) isolated from cow rumen.

**GenBank accession number:** GCF_900103415 (genome); AY196673 (16S rRNA).

### *Methanocatella oralis* (Ferrari *et al*. 1995) Protasov and Brune comb. nov.

**Etymology**: o.ra'lis. L. neut. n. *os*, mouth; N.L. fem. adj. *oralis*, pertaining to the mouth.

**Basonym:** *Methanobrevibacter oralis* Ferrari *et al*. 1995

**Synonym:** *‘Methanocatella oralis’* Protasov and Brune 2023 (not validly published).

**Description:** The description remains the same as in Miller [40]. Includes all genomes with ≥95% average nucleotide identity to the genome of the type strain. The type strain has a genome size of 2.1 Mbp and a G+C content of 27.7 mol%.

**Type strain:** ZR^T^ (DSM 7256, JCM 30027) isolated from the oral cavity of a human.

**GenBank accession number:** GCF_001639275 (genome); HE654003 (16S rRNA).

### *Methanocatella thaueri* (Miller and Lin 2002) **Protasov and Brune** comb. nov.

**Etymology:** thau'er.i. N.L. gen. masc. n. *thaueri*, of Thauer, named in honour of Rolf K. Thauer for his fundamental contributions to the delineation of the biochemistry of methanogenesis.

**Basonym:** *Methanobrevibacter thaueri* Miller and Lin 2002.

**Synonym:** *‘Methanocatella thaueri’* Protasov and Brune 2023 (not validly published).

D**escription:** The description remains the same as in Miller and Lin [41]. Includes all genomes with ≥95% average nucleotide identity to the genome of the type strain. The type strain has a genome size of 2.2 Mbp and a G+C content of 36.9 mol%.

**Type strain:** CW^T^ (DSM 11955, JCM 39455) isolated from cow faeces.

**GenBank accession number:** GCF_003111625 (genome); U55236 (16S rRNA).

### *Methanocatella woesei* (Miller and Lin 2002) Protasov and Brune comb. nov.

**Etymology:** woe’se.i. N.L. gen. masc. n. *woesei*, of Woese, named in honour of Carl R. Woese for his pioneering contributions to the understanding of the phylogeny of methanogens and other micro-organisms.

**Basonym:** *Methanobrevibacter woesei* Miller and Lin 2002.

**Synonym:** *‘Methanocatella woesei’* Protasov and Brune 2023 (not validly published)

**Description:** The description remains the same as in Miller and Lin [41]. Includes all genomes with ≥95% average nucleotide identity to the genome of the type strain. The type strain has a genome size of 1.5 Mbp and a G+C content of 29.9 mol%.

**Type strain:** GS^T^ (DSM 11979, JCM 39456) isolated from goose faeces.

**GenBank accession number:** GCF_003111605 (genome); U55237 (16S rRNA).

### *Methanoflexus* Protasov and Brune gen. nov.

**Etymology:** Me.tha.no.fle'xus. N.L. neut. n. *methanum*, methane; N.L. pref. *methano-*, pertaining to methane; L. masc. n. *flexus*, a bending, turning, winding; N.L. masc. n. *Methanoflexus*, methane-producing (organism) with a curved shape.

**Synonym:** *‘Methanoflexus’* Protasov and Brune 2023 (not validly published).

**Description:** Members of the genus form a monophyletic group defined by both phylogenomic analysis and its relative evolutionary divergence from other members of the family *Methanobrevibacteraceae*. Cells are curved rods that occur singly, in pairs or in short chains. Cells stain Gram-positive. Non-motile. Strictly anaerobic. Use H_2_ + CO_2_ as substrates for methanogenesis. Optimum growth temperature is 30 °C. Require media supplemented with yeast extract or rumen fluid. Genome size ranges from 2.47 to 3.26 Mbp; G+C content from 24.0 to 25.7 mol%.

**Type species:** *Methanoflexus curvatus*

**Parent taxon:** *Methanobrevibacteraceae* Protasov and Brune

### *Methanoflexus curvatus* (Leadbetter and Breznak 1997) Protasov and Brune comb. nov.

**Basonym:** *Methanobrevibacter curvatus* Leadbetter and Breznak 1997.

**Synonym:** *‘Methanoflexus curvatus’* Protasov and Brune 2023 (not validly published).

**Etymology:** cur.va'tus. L. masc. part. adj. *curvatus*, bent, curved; referring to the shape of the cell.

**Description:** The species description remains the same as in Miller [40]. Includes all genomes with ≥95% average nucleotide identity to the type genome. The type strain has a genome size of 2.4 Mbp and a G+C content of 25.7 mol%.

**Type strain:** RFM-2^T^ (DSM 11111, JCM 39452) isolated from the termite *Reticulitermes flavipes*.

**GenBank accession number:** GCF_001639295 (genome); U62533 (16S rRNA).

### *Methanomonile* Protasov and Brune gen. nov.

**Etymology:** Me.tha.no.mo.ni'le. N.L. neut. n. *methanum*, methane; N.L. pref. *methano-*, pertaining to methane; L. neut. n. *monile*, a necklace; N.L. neut. n. *Methanomonile*, a methane-producing chain, referring to the short chains of cells characteristic of this genus.

**Description:** Members of the genus form a monophyletic group defined by both phylogenomic analysis and its relative evolutionary divergence from other members of the family *Methanobrevibacteraceae*. Cells are cocci; they occur singly, in pairs or in chains. Cells stain Gram-positive. Non-motile. Strictly anaerobic. Use H_2_ + CO_2_ as substrates for methanogenesis. Require complex media supplemented with acetate, amino acids, vitamins and yeast extract. Optimum growth temperature is 35–42 °C. Genome size ranges from 1.56 to 1.93 Mbp; G+C content from 26.1 to 27.2 mol%.

**Type species:** *Methanomonile shimae*

**Parent taxon:** *Methanobrevibacteraceae* Protasov and Brune

### *Methanomonile shimae* Protasov and Brune sp. nov.

**Etymology:** shi'mae. N.L. gen. masc. n. *shimae*, of Shima, named in honour of Seigo Shima for his notable contributions to the understanding of the biochemistry of methanogenesis.

**Description:** Cells are cocci, 0.5–2 µm in diameter; they occur singly, in pairs, or in chains. Cells stain Gram-positive. Non-motile. Strictly anaerobic. Optimum growth temperature is 34 °C. Use H_2_ + CO_2_ as substrates for methanogenesis; H_2_ can be substituted with formate. Require complex medium supplemented with acetate, amino acids, vitamins and yeast extract. Includes all genomes with ≥95% average nucleotide identity to the genome of the type strain. The type strain has a genome size of 1.93 Mbp and a G+C content of 26.4 mol%.

**Type strain:** Ec1^T^ (DSM 116169, JCM 39518) isolated from the cockroach *Ergaula capucina*.

**GenBank accession number:** GCF_046197865 (genome); PQ643846 (16S rRNA).

### *Methanothermobaculaceae* Protasov and Brune fam. nov.

Etymology: *Methanothermobaculaceae* (Me.tha.no.ther.mo.ba.cu.la'ce.ae. N.L. neut. n. *Methanothermobaculum*, type genus of the family; L. fem. pl. n. suff. *-aceae* ending to denote a family. N.L. fem. pl. n. *Methanothermobaculaceae*, the family of the genus *Methanothermobaculum*).

**Description:** Members of the family form a monophyletic group that is defined by phylogenomic analysis and its relative evolutionary divergence from other members of the order *Methanobacteriales*. The family has only one genus, *Methanothermobaculum*.

**Type genus:** *Methanothermobaculum* Protasov and Brune

**Parent taxon:** *Methanobacteriales* Balch and Wolfe 1981

### *Methanothermobaculum* Protasov and Brune gen. nov

**Etymology:** Me.tha.no.ther.mo.ba'cu.lum. N.L. neut. n. *methanum*, methane; N.L. pref. *methano-*, pertaining to methane; Gr. masc. adj. *thermos*, hot; L. neut. n. *baculum*, rod, stick. N.L. neut. n. *Methanothermobaculum*, a methane-producing rod from hot environments.

**Description:** Members of the genus form a monophyletic group defined by both phylogenomic analysis and its relative evolutionary divergence from other members of the family *Methanothermobaculaceae*. Phenotypic properties are the same as for the type species.

**Type species:** *Methanothermobaculum tenebrarum*

**Parent taxon:** *Methanothermobaculaceae* Protasov and Brune

### *Methanothermobaculum tenebrarum* (Nakamura *et al*. 2013) Protasov and Brune comb. nov.

**Etymology:** te.ne.bra'rum. L. gen. pl. n. *tenebrarum*, of darkness, of a dark place, referring to its habitat, the deep terrestrial subsurface.

**Basonym:** *Methanothermobacter tenebrarum* Nakamura *et al*. 2013.

**Description:** The description remains the same as in Nakamura *et al*. [45]. Includes all genomes with ≥95% average nucleotide identity to the genome of the type strain. The type strain has a genome size of 1.49 Mbp and a G+C content of 42.0 mol%.

**Type strain:** RMAS^T^ (DSM 23052, JCM 16532) isolated from formation water of a natural gas well.

**GenBank accession number:** GCF_023167465 (genome); AB523785 (16S rRNA).

## Supplementary material

10.1099/ijsem.0.007286 Supplementary Material 1.

10.1099/ijsem.0.007286 Supplementary Material 2.

## Abbreviations

GTDB: Genome Taxonomy Database
ICNP: International Code of Nomenclature of Prokaryotes
MAG: metagenome-assembled genome
RED: relative evolutionary divergence
SeqCode: Code of Nomenclature of Prokaryotes Described from Sequence Data
SH-aLRT: Shimodaira–Hasegawa approximate-likelihood ratio test
