# Supplementary material for: Taxonomic revision of the genus Methanobrevibacter, description of Methanomonile shimae gen. nov. sp. nov., and proposal of Methanobrevibacteraceae fam. nov
Source: Int J Syst Evol Microbiol. 2026 Sep 15;76(9):007286. doi: 10.1099/ijsem.0.007286 (PMC13585281; doi:10.1099/ijsem.0.007286)
Supplement: Supplementary file 1 [file ijsem-76-07286-s001.pdf]

Supporting Information

Supplementary Figures

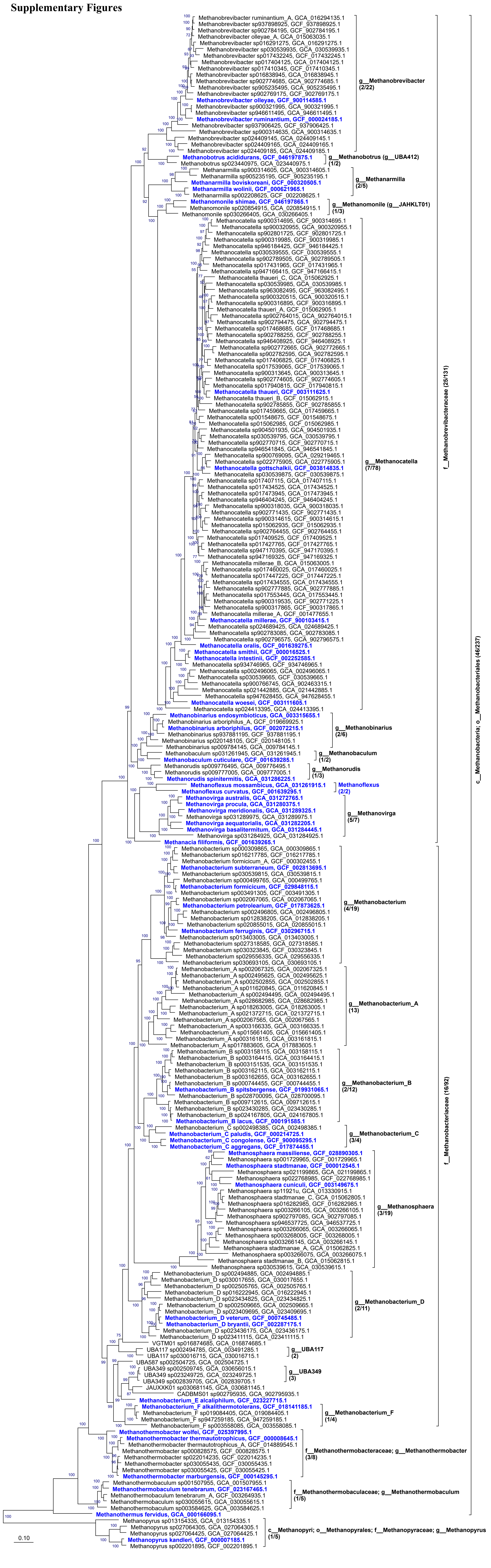

Fig. S1. Phylogenomic tree of all species-level lineages in the order *Methanobacteriales* recognized in GTDB. Type species are shown in blue. The maximum-likelihood tree was inferred from the concatenated alignment of 53 protein-coding genes using IQ-tree; bullets on the internal nodes indicate UFBoot support (● ≥ 99%, 1000 replicates). Members of *Methanopyri* were used as outgroup.

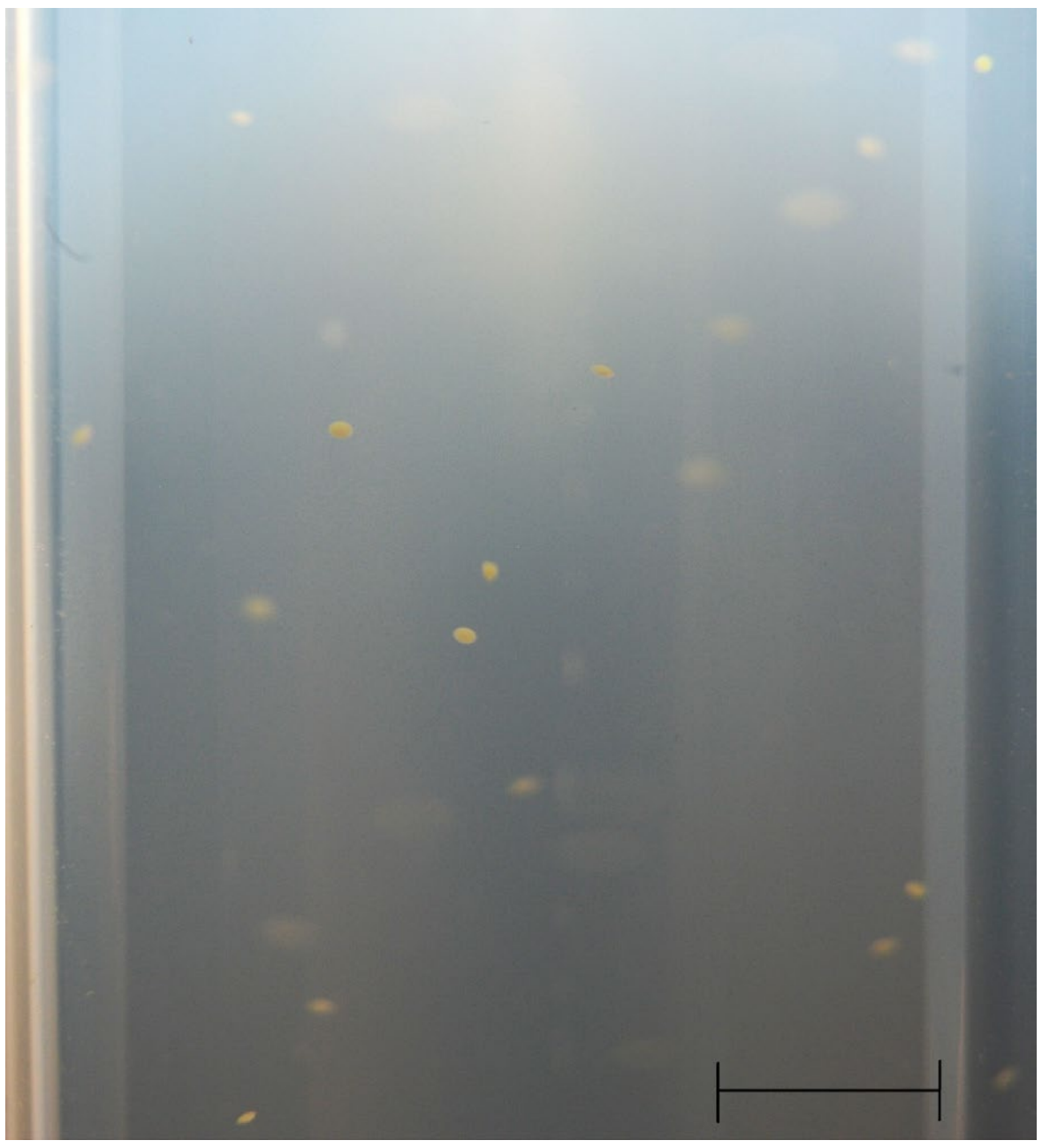

Fig. S2. Colonies of *Methanomonile shimae* Ec1^{T} in a deep-agar dilution tube, two weeks after inoculation (scale bar, 10 mm).

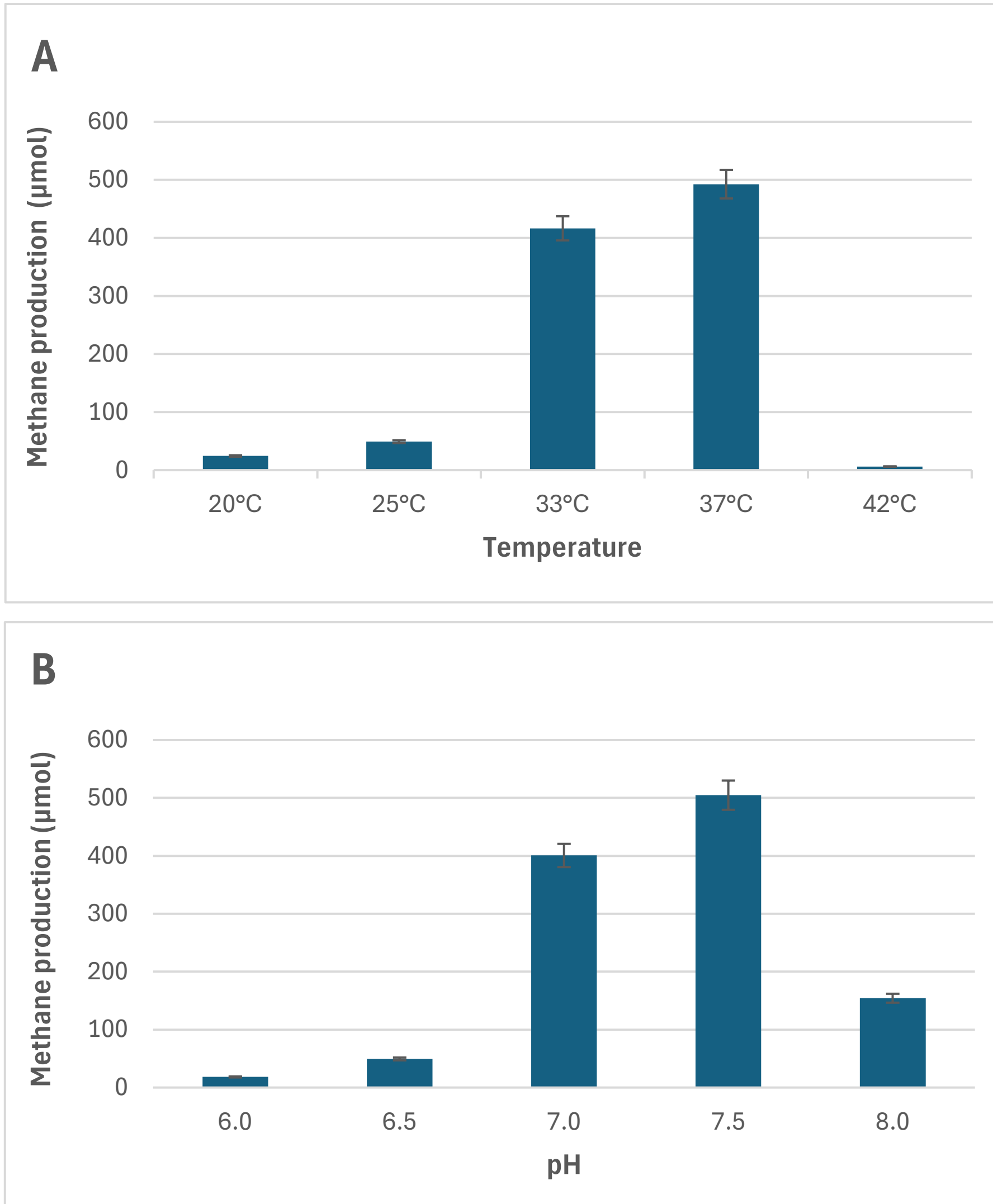

Fig. S3. Methane formation in 5-ml cultures of *Methanomonile shimae* strain Ec1 at different temperatures (A) and pH (B) after 7 d of incubation.
